# Refractory Nontyphoidal Salmonella Empyema Necessitans in a Man Living With HIV: A Case Report and Review of the Literature

**DOI:** 10.1155/crdi/8859848

**Published:** 2025-10-23

**Authors:** Dillon Guo Dong Yeo, Wui Mei Chew, Felicia Teo, Qin Yong See, Aza Abdulmawjood Taha, Edwin Chong Yu Sng

**Affiliations:** ^1^Department of Infectious Diseases, Changi General Hospital, Singapore; ^2^Department of Respiratory and Critical Care Medicine, Changi General Hospital, Singapore; ^3^Department of Radiology, Changi General Hospital, Singapore; ^4^Department of Care and Health Integration, Changi General Hospital, Singapore

## Abstract

Empyema necessitans is a rare complication of untreated/undertreated empyema characterized by the invasion and progression of empyema beyond the pleura into the chest wall. With the availability of antibiotics, empyema necessitans is now rarely seen in clinical practice. Effective treatment of empyema necessitans entails aggressive source control with surgical decortication in conjunction with prolonged culture-directed antibiotics. The most common etiologies include *Mycobacterium tuberculosis*, *Actinomyces*, *Staphylococcus aureus*, and *Streptococcus* specie*s.* Here, we report a case of refractory nontyphoidal *Salmonella* empyema necessitans in a patient who is virologically suppressed on HIV treatment and highlight the challenges in management when surgical decortication could not be performed.

## 1. Background

Empyema necessitans is a rare complication of empyema, characterized by progression of an empyema beyond the pleural cavity into the skin and soft tissue of the chest wall. It typically occurs when empyema is left undiagnosed or improperly treated. Its incidence has fallen with the invention of antibiotics. The most frequently reported etiology is *Mycobacterium tuberculosis* [1–3]*. Actinomyces* species [4], *Staphylococcus aureus* [5], *Streptococcus milleri* [6], *Streptococcus pneumoniae* [2], and anaerobes [7] have also been identified as causes of empyema necessitans. Here, we present a rare case of refractory empyema necessitans due to *Salmonella enterica* serovar Brancaster in a patient who is virologically suppressed on HIV treatment.

## 2. Case Presentation

A 52-year-old male of Southeast Asian descent, living with human immunodeficiency virus (HIV), presented to our emergency department with one week of fever, cough, and breathlessness. His antiretroviral therapy was dolutegravir 50 mg once daily and lamivudine 300 mg once daily. While he was virologically suppressed for the past 7 years, CD4 recovery was poor with CD4 count ranging between 100 cells/mm^3^ (15.6%) and 129 cells/mm^3^ (18.7%). The poor CD4 recovery was attributed to late presentation of HIV, with a CD4 count of 24 cells/mm^3^ (6.1%) at diagnosis. He also suffered from bilateral trapped lungs from previous empyemas caused by Group G *Streptococcus* and *Mycobacterium avium complex* at the time of HIV diagnosis. This is complicated by chronic Type 2 respiratory failure requiring long-term home ventilation. He takes trimethoprim-sulfamethoxazole 480 mg once daily for prophylaxis of *Pneumocystis jiroveci* pneumonia.

**Figure 1:**
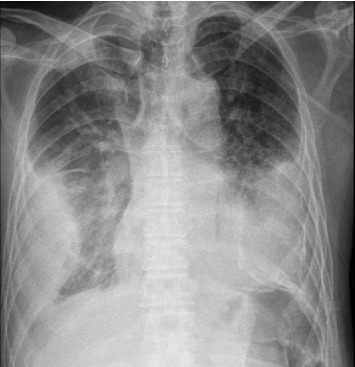
Chest x-ray at initial presentation demonstrating bilateral chronic pleural effusions.

**Figure 2:**
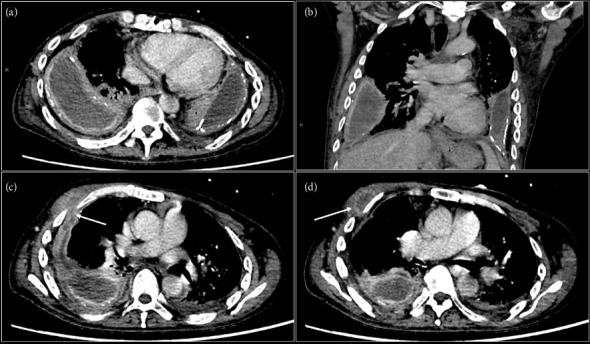
Contrast-enhanced computed tomographic images of the thorax in the portal venous phase, in axial (a) and coronal (b) planes, demonstrating the presence of bilateral pleural collections with thickened pleura and calcifications, which are features of chronic empyema. Consolidations are also seen in the lower lobes bilaterally. Axial images at different levels show the presence of a fistulous tract extending anteriorly from the right empyema (arrow, (c)), leading to a thick-walled collection in the right chest wall (arrow, (d)).

At presentation, he was febrile (temperature 38.4 degrees Celsius). Blood pressure was 97/63 mmHg, and heart rate was 122 beats per minute. Oxygen saturations were 96% on 4 L/min of supplementary oxygen. He was cachectic with a body mass index of 15. He appeared lethargic with labored breathing. Auscultation revealed decreased air entry over bilateral lung bases, with coarse crepitations heard over the right hemithorax. The rest of the physical examination was unremarkable. Initial laboratory investigations revealed an elevated total white cell count (14.9 × 10^3^/μL) and elevated C-reactive protein (193.5 mg/L). An arterial blood gas showed acute-on-chronic worsening of Type 2 respiratory failure. Chest radiograph showed stable bilateral pleural effusions, with increased opacification in both lung fields (Figure 1). A contrast-enhanced computed tomography (CECT) scan of the thorax demonstrated stable moderate bilateral loculated pleural collections, larger on the right, of raised attenuation with enhancing pleural thickening and scattered pleural calcifications suggestive of chronic empyemas. The right-sided pleural collection appeared to communicate with a right anterior chest wall rim-enhancing collection via a thin tract between the right anterior 3^rd^ and 4^th^ rib (Figure 2). There was no bacterial growth on three sets of blood cultures performed.

He was diagnosed with empyema necessitans. Thoracocentesis was performed, and a 12-French chest tube was inserted into the right pleural cavity. Blood-stained purulent fluid was aspirated. Cultures of the pleural fluid grew serogroup B *Salmonella*, which was eventually identified to be *Salmonella enterica* serovar Brancaster (serotyped and classified according to the White–Kauffman–Le Minor scheme). Antimicrobial susceptibility testing revealed that the isolate was susceptible to ceftriaxone and meropenem, intermediate to ciprofloxacin, and resistant to ampicillin and sulfamethoxazole-trimethoprim (Table 1). The minimum inhibitory concentration of azithromycin was 4 mg/L (no clinical breakpoint for nontyphoidal *Salmonella*). Intravenous ceftriaxone 2 g daily was commenced, but this was subsequently escalated to meropenem 1 g every 8 h due to breakthrough fevers while on ceftriaxone. He was referred to thoracic surgery for consideration of surgical decortication. Due to his frailty, low body mass index of 15, and chronic Type 2 respiratory failure necessitating long-term oxygen therapy and nocturnal noninvasive ventilation, both the anesthetist and thoracic surgeon assessed that he was at high risk of perioperative complications, with either one-lung ventilation or conventional bilateral ventilation. As source control with surgical decortication was not feasible, the chest tube was kept to facilitate continuous drainage of the infected pleural fluid, while intravenous antibiotic was continued. At 5 weeks postinsertion, drain output had decreased significantly, prompting removal of the chest tube. At this stage, he was clinically well without fever. Due to concerns of relapse due to inadequate source control, he was discharged on long-term suppressive therapy with oral azithromycin 500 mg daily.

### 2.1. First Relapse

Around 8 weeks after discharge, our patient returned to the emergency department with fever and breathlessness. Laboratory investigations revealed raised total white cell count (12.0 × 10^3^/μL) and elevated C-reactive protein (138.3 mg/L). Thoracocentesis of the right pleural effusion yielded pus which grew again serogroup B *Salmonella.* Susceptibility testing revealed that the MIC of azithromycin had increased to 64 mg/L. He was started on a higher dose of intravenous ceftriaxone 3 g daily, to which the *Salmonella* remained susceptible (ceftriaxone MIC 0.5 mg/L). Thoracic surgery was again consulted for surgical decortication, but surgical risks remained prohibitive due to his poor pulmonary function and overall frailty. During his treatment, pleural fluid was repeatedly sampled for cultures. Pleural fluid was only sterilized after close to 3 months of high-dose intravenous ceftriaxone treatment and drainage via chest tube. He was clinically well without fever but C-reactive protein, which improved from an initial 138 mg/L, remained elevated at around 50 mg/L despite 3 months of antibiotics. A joint decision among the infectious diseases team, respiratory medicine team, and the patient was made to discontinue antibiotics but to leave the chest tube in indefinitely to allow continued drainage of the pleural fluid as there was likely residual infection without surgical decortication. The patient was taught and trained on how to care for the drain while at home.

### 2.2. Second Relapse

**Figure 3:**
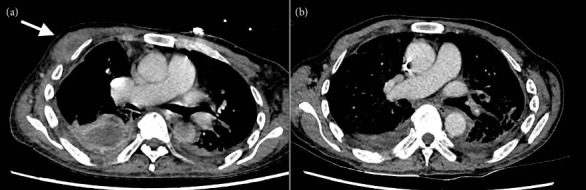
Contrast-enhanced computed tomographic images of the thorax in the portal venous phase, in axial planes. The thick-walled collection in the right chest wall (arrow, (a)) observed at presentation (April 2024) is no longer seen on the latest computed tomography scan (b) (January 2025). There is also significant improvement in the right pleural collection.

**Figure 4:**
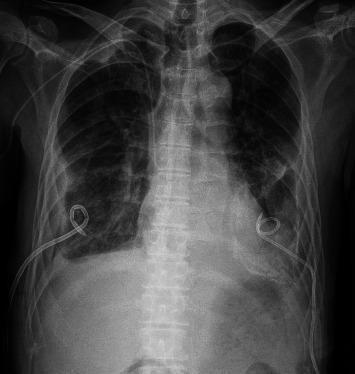
Chest x-ray prior to discharge after the patient's 2^nd^ relapse, demonstrating the interval improvement of bilateral pleural effusions.

Two months after cessation of antibiotics, he was readmitted as the pleural fluid from the right chest drain was becoming more purulent and C-reactive protein had risen to 145 mg/L. Culture of the right pleural fluid again grew serogroup B *Salmonella*, which remained susceptible to ceftriaxone and meropenem. The MIC of azithromycin for this isolate had decreased from 64 to 16 mg/L. Repeat CECT of the thorax demonstrated significant decrease in size of right empyema, with resolution of the right anterior chest wall collection (Figure 3). Aspiration of the chronic left pleural effusion yielded stale blood, but culture did not grow any organism. He was initiated on a combination of two antibiotics, intravenous ceftriaxone 2 g every 12 h and oral azithromycin 500 mg once daily. Due to the refractoriness of the infection despite prolonged courses of antibiotics and ongoing chest tube drainage, the patient was counseled on the need for long-term, potentially life-long, intravenous ceftriaxone, and oral azithromycin, with indefinite chest drainage. Repeat chest radiograph showed interval decrease in sizes of bilateral pleural effusions (Figure 4). Prior to discharge, the left drain was removed, and he was discharged home with home medical services, with the right pleural drain in situ. At the point of writing this manuscript, he was 5 months into antibiotic treatment and right chest drainage and remained well in the community. C-reactive protein had also improved significantly, ranging around 10 mg/L on weekly checks.

## 3. Discussion

Empyema necessitans is characterized by the invasion and progression of an empyema beyond the pleural cavity, into the skin and soft tissues of the chest wall. Infections due to *Mycobacterium tuberculosis* and *Actinomyces*, the most common etiologic agents, tend to present in an indolent nature. Conversely, infections secondary to pyogenic bacteria such as *S. aureus and S. pneumoniae* may present more fulminantly. As our patient was in septic shock at presentation, pyogenic bacteria were suspected. The isolation of *Salmonella* from pleural fluid was unexpected, as it is an uncommon cause of empyema [8] and an even rarer cause of empyema necessitans. To our knowledge, there have only been two other case reports of *Salmonella* empyema necessitans [9, 10] (Table 2).

*Salmonella* is a gram-negative, facultative anaerobic bacilli of the Enterobacteriaceae family. It is an intracellular pathogen and can be classified into typhoidal or nontyphoidal serotypes. Humans are the only reservoir for the typhoidal serotypes, Typhi and Paratyphi, while animals such as poultry, cattle, and reptiles are the natural reservoirs for nontyphoidal serotypes. Transmission of nontyphoidal *Salmonella* occurs via ingestion of contaminated food or animal contact. *Salmonella enterica* serovar Brancaster, the causative agent for the empyema necessitans in our patient, was nontyphoidal.

Nontyphoidal salmonellosis causes various clinical syndromes, including gastroenteritis, primary bacteremia, endovascular infection, visceral organ abscesses, and osteomyelitis. Cellular immunity plays a significant role in the protection against *Salmonella*, an intracellular pathogen. Immunocompromised states increase the risk for bacteremia and extraintestinal infections [11, 12]. In particular, HIV infection, corticosteroid use, diabetes mellitus, and malignancies [12–14] are risk factors for *Salmonella* infection and severe disease. Even though our patient was virologically suppressed on antiretroviral therapy, his low CD4 count likely predisposed him to *Salmonella* infection. His chronic pleural effusion was likely hematogenously seeded during an episode of occult bacteremia, leading to the development of empyema. Because he was on long-term sulfamethoxazole-trimethoprim for *Pneumocystis jirovecii* pneumonia prophylaxis, we postulate that the presentation could have been delayed due to partial suppression of the infection. Nevertheless, the empyema progressed over time, extending beyond the pleural space into the chest wall, resulting in empyema necessitans. Among the other two cases of *Salmonella* empyema necessitans in the literature, one occurred in a patient with poorly controlled diabetes mellitus with a glycated hemoglobin of 12.4% [9] and the other occurred in a patient with resected glioblastoma on radiotherapy [10].

Preferred antibiotics for the treatment of nontyphoidal *Salmonella* include a fluoroquinolone such as ciprofloxacin or a third-generation cephalosporin such as ceftriaxone, given the high tissue and intracellular concentrations of these agents [14]. However, strains with multiple antibiotic resistances, such as those expressing extended-spectrum beta-lactamases [15, 16] have been reported. In our case, the *Salmonella* was only susceptible to ceftriaxone and meropenem, necessitating intravenous administration of antibiotics and limiting our options for suppressive therapy. Due to the spread of resistant strains of *Salmonella*, empirical antibiotic choice should be based on local antibiograms.

Azithromycin, a macrolide antibiotic, has been demonstrated to accumulate at significant concentrations intracellularly, offering an alternative oral agent against *Salmonella* [17]. Both the Clinical and Laboratory Standards Institute (CLSI) and European Committee on Antimicrobial Susceptibility Testing (EUCAST) recommend a susceptibility breakpoint of ≤ 16 μg/mL to distinguish between sensitive and resistant strains of *Salmonella typhi* [18]. At present, there are no CLSI or EUCAST breakpoints for nontyphoidal *Salmonella* species. However, studies demonstrate in vitro activity against these strains [19], with microbiological studies supporting the use of an epidemiological cutoff value of ≤ 16 μg/mL for wild-type isolates [20]. While human studies are lacking, an animal study demonstrated excellent penetration of azithromycin into the pleural fluid, with pleural fluid concentrations exceeding those in serum after intravenous administration [21]. Given the above and the lack of an alternative oral agent, we opted to use azithromycin to suppress residual infection after completion of intravenous antibiotics.

Despite having received prolonged courses of culture-directed intravenous antibiotics, achieving microbiological cure was challenging. The first relapse occurred while on oral azithromycin, and the second relapse occurred despite ongoing drainage of the infected pleural fluid via a chest tube. Apart from antibiotics, effective treatment of empyema requires adequate source control, which often includes surgical decortication [9, 10]. Without adequate source control, the likelihood of treatment failure or relapse is high. Notably, both patients with *Salmonella* empyema necessitans in the literature underwent decortication and survived. Unfortunately, the risks for surgical decortication were too high in our patient. With limited treatment options, he was agreeable to long-term chest tube drainage and a prolonged, potentially life-long course of intravenous antibiotics via home medical services. With such a treatment strategy, he has remained clinically well in the community for the past 5 months. Recently, phage therapy has been used successfully in the treatment of empyema due to multidrug resistant *Pseudomonas aeruginosa* and may offer a potential treatment option [22, 23].

In conclusion, we presented a rare case of refractory *Salmonella enterica* serovar Brancaster empyema necessitans in a patient virologically suppressed on HIV treatment. Although rare, nontyphoidal *Salmonella* should be considered as an etiological agent for empyema necessitans in patients who are immunocompromised. Management was challenging as definitive source control with surgical decortication could not be performed. With two relapses and limited treatment options, our patient was managed with long-term chest tube drainage and a prolonged, potentially life-long, course of intravenous antibiotics and has remained clinically well 5 months into treatment.

## Figures and Tables

**Table 1:** Antimicrobial susceptibilities of *Salmonella enterica* serovar Brancaster.

Antibiotics	Episode						
	Susceptibility/minimum inhibitory concentration (mg/L)						
	Initial presentation		1^st^ relapse		2^nd^ relapse		CLSI breakpoints
Amikacin	R	—	R	—	R	—	No clinical breakpoint
Ampicillin	R	—	R	—	R	—	≤ 8
Sulfamethoxazole-trimethoprim	R	—	R	—	R	—	≤ 2/38
Ceftriaxone	S	0.25	S	0.5	S	0.5	≤ 1
Meropenem	S	0.06	S	0.25	S	0.25	≤ 1
Ciprofloxacin	I	0.5	R	1	R	1	≤ 0.06
Azithromycin	No clinical breakpoint	4	No clinical breakpoint	64	No clinical breakpoint	16	No clinical breakpoint

*Note:* R = resistant, S = sensitive, and I = intermediate. The susceptible breakpoint for azithromycin and *Salmonella typhi* is ≤ 16 mg/L. No clinical breakpoints are available for nontyphoidal species. The Clinical and Laboratory Standards Institute (CLSI) breakpoints are referenced from the CLSI M100 Performance Standards for Antimicrobial Susceptibility Testing.

**Table 2:** Summary of case reports describing *Salmonella* as a cause for empyema necessitans.

Reference, location	Age	Past medical history	Pathology	Microbiology	Treatment	Outcome
Dey et al. [9], Bangalore, India	44	Type 2 diabetes mellitus	Left empyema with anterior chest wall collection	*Salmonella enterica* serotype typhi	Thoracocentesis, decortication, and left lower lobectomy. IV ceftriaxone, oral cefixime	Survived
Urlapu et al. [10], Bronx, USA	57	Resected glioblastoma on radiation therapy	Left empyema with sternoclavicular joint septic arthritis, infraclavicular collection	*Salmonella enterica* species	Thoracocentesis, video-assisted thoracic surgery with decortication. IV ceftriaxone.	Survived
Present case	52	Virologically suppressed HIV, with immunological nonresponse	Right empyema necessitans with extension into the anterior chest wall	*Salmonella enterica* serovar Brancaster	Repeated thoracocentesis with continued antibiotic therapy	Survived
